# Exploring the significance of morphological diversity for cerebellar granule cell excitability

**DOI:** 10.1038/srep46147

**Published:** 2017-04-13

**Authors:** Catriona M. Houston, Efthymia Diamanti, Maria Diamantaki, Elena Kutsarova, Anna Cook, Fahad Sultan, Stephen G. Brickley

**Affiliations:** 1 Departent of Life Sciences, Imperial College London, UK; 2 Neuroscience, Physiology & Pharmacology, University College London, UK; 3 Werner-Reichardt Centre for Integrative Neuroscience, Tübingen, Germany; 4 Department of Neurology and Neurosurgery McGill University, Montréal, Canada; 5 Department Cognitive Neurology, Hertie-Institute for Clinical Brain Research, University of Tübingen, Germany; 6 Department Integrative medical biology, Umeå University, Sweden; 7 Centre for Neurotechnology, Imperial College London, UK

## Abstract

The relatively simple and compact morphology of cerebellar granule cells (CGCs) has led to the view that heterogeneity in CGC shape has negligible impact upon the integration of mossy fibre (MF) information. Following electrophysiological recording, 3D models were constructed from high-resolution imaging data to identify morphological features that could influence the coding of MF input patterns by adult CGCs. Quantification of MF and CGC morphology provided evidence that CGCs could be connected to the multiple rosettes that arise from a single MF input. Predictions from our computational models propose that MF inputs could be more densely encoded within the CGC layer than previous models suggest. Moreover, those MF signals arriving onto the dendrite closest to the axon will generate greater CGC excitation. However, the impact of this morphological variability on MF input selectivity will be attenuated by high levels of CGC inhibition providing further flexibility to the MF → CGC pathway. These features could be particularly important when considering the integration of multimodal MF sensory input by individual CGCs.

A strategic objective for the neuroscience community is to reach a comprehensive understanding of brain circuits that links anatomy and neuronal dynamics to behaviour. As cerebellar granule cells (CGCs) receive a small number of synapses onto relatively simple dendrites, they offer a unique opportunity to fully understand synaptic integration within a single neuronal type that participates in well described circuit behaviour; namely the relay of mossy fibre (MF) sensory input to cerebellar Purkinje cells. However, many important aspects of CGC basic anatomical connectivity have simply not been adequately described hindering a full description of the cerebellar cortex. The ability of CGCs to encode MF information during motor learning^1^,^2^,^3^ is believed to involve activity-dependent alterations in the strength of the parallel fibre synapses that a Purkinje cell receives from ~200,000 independent CGCs^4^. Long-term changes in the strength of MF → CGC connections have been reported^5^,^6^,^7^ but, at an anatomical level, the MF → CGC projection appears hard-wired with no ability to alter connectivity following changes in sensory input^8^. Alterations to short-term dynamics at MF → CGC synapses^9^,^10^, as well as changes in membrane excitability^11^,^12^,^13^, offer the capacity to modulate the transfer of MF information. For example, theoretical models of the cerebellar circuit describe how sparse coding of MF inputs is influenced by the level of inhibition^3^,^14^,^15^,^16^. However, the small size of CGCs underlies a commonly held assumption that individual MF inputs arriving onto each dendrite claw will have an equal impact on CGC excitability^14^,^17^. This study considers, for the first time, how differences in CGC shape could influence the representation of MF sensory information within the cerebellar circuit.

CGCs are morphologically simple and are also considered one of the simplest cell types in terms of connectivity^18^. Electrophysiological studies have demonstrated the variability in short-term synaptic plasticity that exists at individual MF → CGC synapses^19^,^20^,^21^, and have highlighted the importance of controlling intrinsic CGC excitability for gain control and sparse coding of MF inputs^11^,^13^,^22^. However, quantitative information on CGC morphology has been lacking due to the technical challenge of obtaining high-resolution morphological data from this small cell type.

We have generated a sufficient quantity of individually labelled CGCs to generate high resolution 3D models and identify a number of previously unappreciated morphological features that could impact on the ability of CGCs to represent information arriving from the MF projection. We demonstrate how the number of potential MF inputs connected to a single CGC is enhanced by dendrite branching. Importantly, by quantifying CGC dendrite length and measuring the distance between claws, we present evidence that CGCs are likely to be connected to the same MF input more than once. A direct quantification of the distance between MF rosettes for individual MF inputs supports this conclusion. We also show that the dendrite closest to the axon has the largest surface area associated with its dendrite claw. By incorporating these features into multi-compartmental models we describe how CGC dendrite length, size and location could influence the MF representation in the cerebellum and propose a novel mechanism for recoding this MF input involving realistic changes in CGC intrinsic properties.

## Results

### The influence of CGC morphological diversity on MF-evoked action potential timing

**Figure 1:**
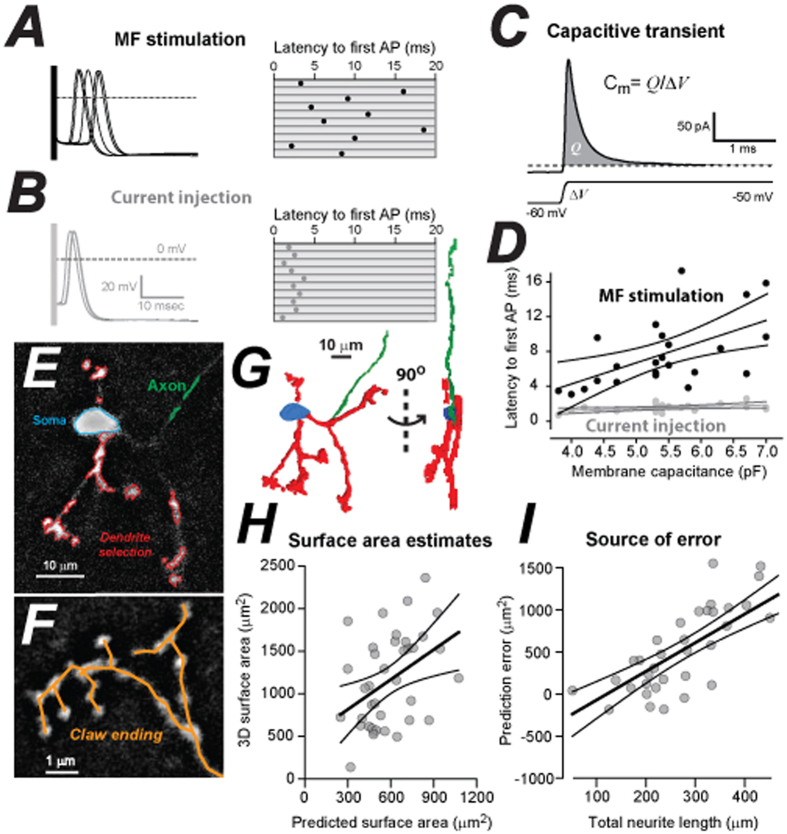
Evidence that cell size influences MF → CGC precision. (**A**) Whole-cell current-clamp recording of APs generated following electrical stimulation of MF inputs onto a CGC in an acute slice preparation of the adult cerebellum. A series of voltage traces are superimposed and the latency to first AP is shown on the right hand raster plot for the first 10 responses. Failures have not been included. (**B**) Superimposed voltage traces of APs elicited following brief 1ms somatic current injection are shown for the same CGC as in (**A**). Note the longer latency and greater variability of APs elicited following MF stimulation compared to somatic current injection. (**C**) The whole-cell voltage-clamp configuration was used to generate an average current trace in response to a 10 mV step in the command voltage. The charge transfer of the brief capacitive transient (grey area) was used to calculate the membrane capacitance and to predict the total membrane surface area for each CGC. (**D**) Scatter plot of membrane capacitance versus mean latency to first AP for MF stimulation (black fills) and somatic current injection (grey fills). The linear regression and 95% confidence levels of the fit are shown. (**E**) A single confocal section of an adult CGC filled with biocytin following whole-cell recording, illustrating the use of a wild-fire algorithm to automatically detect the fluorescent signal associated with dendrites (red), soma (blue) and axon (green) for a single CGC. The flat area surrounding each detected region was used to estimate the surface area of 3D objects. (**F**) Smaller region of a single optical section showing digits within the claw of a single dendrite. The orange lines show the manual tracing method used to measure dendrite length. (**G**) A fully reconstructed 3D model generated from the CGC data shown in (**E**). This model was used to estimate the total surface area of the soma (blue), dendrites (red) and axon (green). (**H**) A scatter plot of the measured surface obtained from all 3D models versus the predicted surface area calculated from the capacitance current transient recorded from the same CGC. Superimposed upon this scatter plot are the linear regression and the 95% confidence levels. (**I**) A scatter plot of the prediction error (3D surface area minus the predicted surface area) as a function of the total neurite length (total dendrite length plus the total axon length) along with the linear regression and the 95% confidence levels for this fit.

To explore the possibility that CGC morphology could influence the integration of MF inputs we systematically compared action potential (AP) timing following current injection into the soma with AP timing following extracellular stimulation of the MF inputs onto individual CGCs. The probability of observing an AP in response to brief somatic current injection (0.8 ± 0.0004; *n* = 23) and MF stimulation (0.8 ± 0.0008; *n* = 23) was similar. However, AP timing was very different (Fig. 1A and B). Following somatic current injection, the mean latency to the first AP was 1.5 ± 0.09 ms with little variation between CGCs (CV = 0.29). Following MF stimulation, the mean latency for the first evoked AP was considerably delayed at 7.7 ± 0.08 ms (paired Wilcoxon signed-rank test, p = 0.00002) and there was greater variability between CGCs (CV = 0.52). The increased latency was to be expected given the proximity of the stimulating electrode (3–5 mm) relative to the recorded cell but, the greater variability observed in AP timing following MF stimulation could indicate diversity at individual MF → CGC connections. Regression analysis indicated a relationship between CGC membrane capacitance (Fig. 1D) and latency to the first AP when considering MF stimulation (r^2^ = 0.29, ANOVA; *P* = 0.005) but this relationship was less pronounced (r^2^ = 0.18, ANOVA; *P* = 0.02) for somatic current injection (Fig. 1D). Therefore, larger CGCs appear to integrate MF inputs more slowly than smaller CGCs indicating that morphological diversity could be impacting on functional diversity to a much greater degree than expected.

### Exploring the ability of CGCs to behave as a single electrical compartment

CGCs are typically described as containing four similar sized dendrites randomly distributed around the soma. Each dendrite is considered to be only 15 μm in length^23^ ending with a claw-like structure that receives synaptic drive from an independent MF rosette. Finally, a single axon leaves the soma and travels towards the molecular layer to form the parallel fibre projection^24^. However, the small size of CGCs has made quantification of these morphological features extremely challenging. In this study, we applied frame averaging and blind deconvolution to improve the signal to noise ratio of individual confocal sections allowing clearer identification of soma (blue), axon (green) and dendrites (red) in each optical section (see Fig. 1E). We then constructed 3D models of individual CGCs (see Fig. 1G). We traced the dendrite and axon through each optical section in order to gain an estimate of the total dendrite and axon length. The example shown in Fig. 1F illustrates the claw-like structures at the end of each dendrite that contains multiple short processes or digits.

To validate the accuracy of the morphological reconstructions we first compared the membrane surface area measurements obtained from 3D reconstructions with biophysical estimates of surface area predicted from the capacitance measurements made from the same cells (see Fig. 1C). Assuming a specific membrane capacitance of 1 μF/cm^2^ 25, our whole-cell measurement of membrane capacitance predicted a lower surface area of 567 ± 30 μm^2^ (*n* = 42) compared to the average value obtained from 3D reconstructions that gave a value of 1154 ± 88 μm^2^ for the same cells. The slope of the linear regression shown in Fig. 1H (1.2 ± 0.4) does provide evidence that surface area measurements obtained following 3D reconstruction will be linearly related to the biophysical estimates predicted from the membrane capacitance measurements, albeit with an overstimation of 463 ± 261 μm^2^ (the y-axis intercept). However, the scatter of data around the linear regression highlights the large cell to cell variability associated with these measurents (r^2^ = 0.15; ANOVA; *P* = 0.008). The voltage-clamp approach will under estimate the total surface area of neurons if certain compartments are electrically isolated from the soma^25^ for example, due to the presence of frequent dendrite branching and/or the presence of high axial resistance compartments (*i.e.* long thin dendrites or axons). To deteremine whether neurite length (dendrite and axon) could contribute to the error associated with the voltage-clamp prediction we estimated the error for each cell (3D surface area minus the predicted surface area). As shown in Fig. 1I, a clear relationship between this prediction error and the total neurite length was suggested by this analysis (r^2^ = 0.56; ANOVA; *P* = 1.2 × 10^−7^) providing a useful validation of the confocal imaging data as a method for measuring membrane surface area and raising the possibility that variability in dendrite/axon morphology could influence MF → CGC integration. In an attempt to explore this possibility we more thoroughly quantified CGC morphology from 3D models.

### Quantification of previously overlooked features of CGC morphology

**Figure 2:**
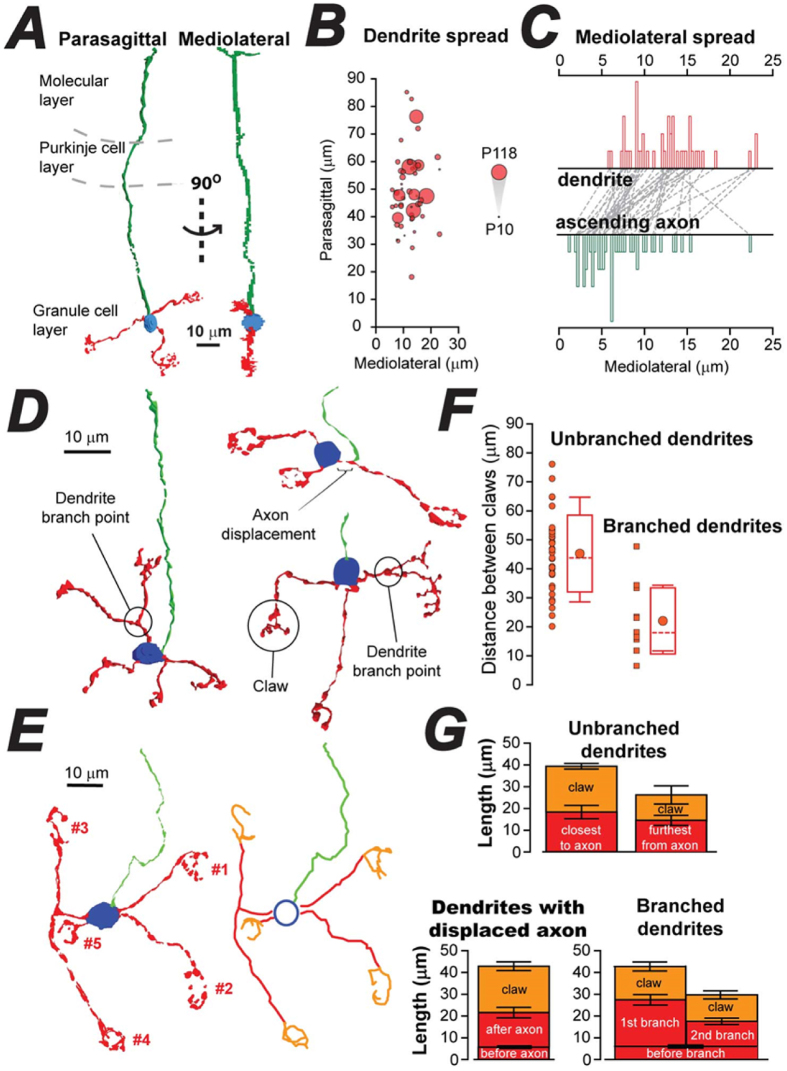
Quantification of CGC features that could impact on timing, coding and integration of MF inputs. (**A**) 3D reconstruction obtained following confocal imaging of an adult CGC filled with biocytin during whole-cell recording. The parasagittal view of this cell (left) has been rotated by 90° to illustrate the narrow mediolateral spread of the axon and dendrites. (**B**) Bubble plot of data obtained for all CGCs plotting the relationship between the mediolateral spread versus the parasagittal spread of the dendrites for all CGCs. The size of the bubble indicates CGC age. (**C**) Histograms of the mediolateral spread for the dendrites (top histogram – red bars) compared to the mediolateral spread for the axon (bottom histogram – green bars). The dashed lines link the individual CGCs from which the latter two measurements were obtained. (**D**) Three representative examples of 3D reconstructions (≥P30) illustrating the presence of dendrite branch points and axon displacement. (**E**) Another example of a 3D reconstruction and the results of manual tracing of dendrites (red) and axon (green) for this cell with the length of the claw digits shown in orange. (**F**) Scatter plot of the Euclidian distance between claws found on unbranched and branched dendrites. The box and whisker plots beside each scatter plot depict the mean (circle), the median (dashed) values for each distribution, the standard deviation (box) and the 10% to 90% quartiles (whisker). (**G**) Bar charts depicting the average length of dendrite within the primary dendrite (red) and also the average length of all the digits within the claw. The top two bar charts compare these distances for unbranched dendrites closest and furthest away from the axon. The bottom left chart show the average values for the dendrite before and after the displaced axon and its associated claw. The right-hand bottom chart illustrates the average dendrite length before and after branch points of the type shown in (**D**). The error bars show the SEM.

**Figure 3:**
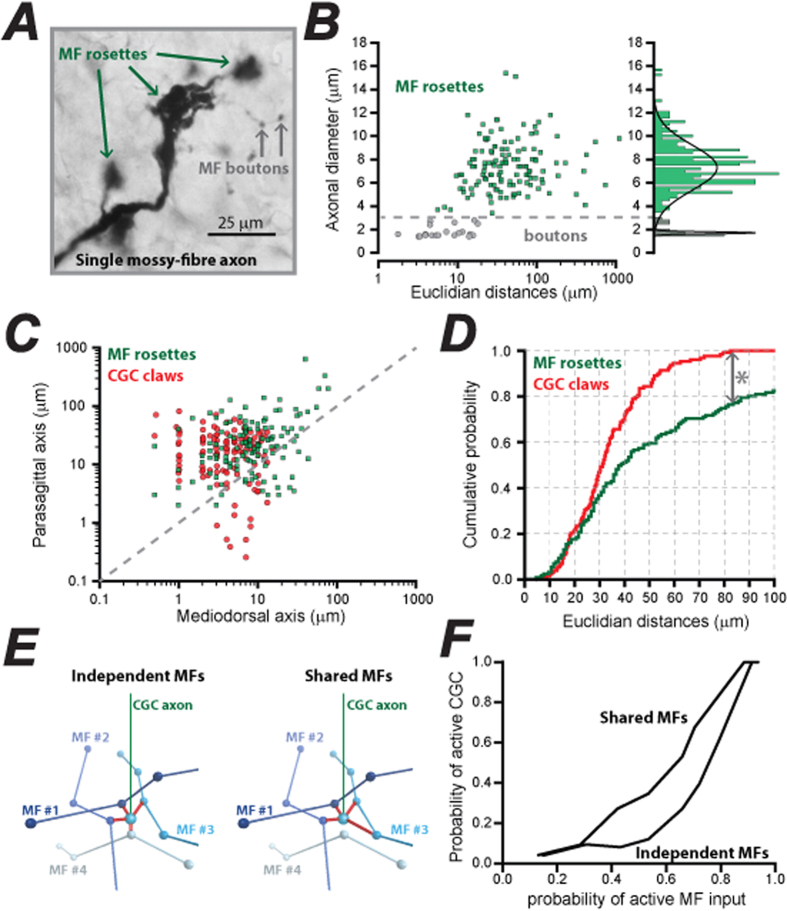
Quantification of MF rosette spacing and comparison with CGC claw spacing. (**A**) Image of a single MF axon containing multiple large rosettes (green arrows) and much smaller swellings thought to be MF boutons (grey arrows). (**B**) Scatter plot of axonal diameter of MF rosettes (green squares) and boutons (grey circles) as a function of Euclidian distance from their nearest neighbour. The histogram on the right of the scatter plot demonstrates how the cut-off for the axonal diameters between rosettes and boutons was determined. The multiple Gaussians that describe the histogram clearly demonstrates the bimodal distribution of axonal diameters. (**C**) Scatter plot of the mediolateral and parasagittal distance between pairs of MF rosettes (green squares) compared to the superimposed scatter plot of the corresponding distances between dendrite claws on each CGC. The dashed line indicates the location of all equidistant points in each axis. Note how the points in both distributions tend to fall above this line indicating greater spread in the parasagittal axis. (**D**) Cumulative probability functions’ of the Euclidian distance between adjacent MF rosettes (green line) and CGC claws (red line). We have not included values above 100 μm to emphasise the span of the CGC claws. Note how this analysis predicts that only ~20% of MF rosettes are beyond the reach of two CGC claws. (**E**) Illustration of the models for independent and shared MFs. The multiple rosettes along a single MF are represented as a single spheres. In the independent MF model shown on the left, a single granule cell with four dendrites (red) that are constrained to be 3.4μm to 27.9 μm in length project in random directions until each dendrite has made contact with one of the randomly placed MFs. However, in this model, each connection must go to an independent MF. On the right single CGC are allowed to receive multiple inputs from a MF as shown for MF #3. (**F**) A plot of the results from the two simulations illustrated in panel E. Each simulation of the model involved a random spike train that provided input to a given proportion of MFs (probability of active MFs). After running simulations of the model for 100 ms, the output was analysed using MATLAB to compare the output of the network (probability of active CGCs). The results from the two models are shown.

3D models revealed several features of CGC morphology that have been overlooked in most models of the cerebellar circuit. For example, it was clear from a 90° rotation of the 3D reconstructions shown in Figs 1G, 2A and 3D that CGC dendrites exhibit restricted mediolateral coverage compared to the dendrite coverage observed in the parasagittal image. This simple observation demonstrates that CGC dendrites are not randomly distributed around the soma. Indeed, on average, the dendrite spread of a typical CGC covers 49.1 ± 1.9 μm (*n* = 51 CGCs) in the parasagittal plane compared to only 12.3 ± 0.6 μm in the mediolateral plane (see Fig. 2B). This anisotropic spatial organisation is very similar to the distribution of rosettes reported for the incoming MF projection^26^. We observed a flat appearance to CGCs at all ages examined. It was also evident in all examples shown that the ascending axon trajectory rarely deviates from the narrow mediolateral spread defined by the CGC dendrites until the split at the T-junction, where the parallel fibres form. To quantify this feature we directly compared the mediolateral spread of the dendrites with the mediolateral spread of the ascending axon for each 3D reconstruction. The ascending axon of the CGC shown in Fig. 2A extended over 200 μm into the molecular layer, but at no point did the axon transect the mediolateral boundary created by the underlying dendrites. On average, the mediolateral spread for the ascending axon was just 6.8 ± 0.6 μm (*n* = 51); nearly half that observed for the corresponding dendrites. It is clear from Fig. 2C that the mediolateral spread associated with the ascending axon is consistently flatter compared to that of the dendrites for virtually all CGCs examined. This feature will have an impact both on the connectivity features of the MF → CGC pathway as well as on the timing of APs arriving along parallel fibres. This feature of CGC anatomy could also be relevant to the organization of cerebellar microzones that are characteristic of CGC connectivity to Purkinje cell clusters^27^.

More features of adult CGCs can be seen in the 3D models illustrated in Fig. 2D and E. The number of MF rosettes potentially connected to a single CGC is expanded by 25% due to dendrite branching. At ≥ P30, the average number of primary dendrites emerging from the soma is 3.9 ± 0.1 (n = 41), yet 40% of mature CGCs are branched (often more than once) such that five MF rosettes will often be connected to a single adult CGC. In 40% of CGCs, we also observed the presence of a displaced axon emerging along a primary dendrite. Figure 2E illustrates the results of the tracing method used for each CGC, with the axon shown in green, the primary dendrites shown in red, and the dendrite associated with the claw shown in orange. We also measured the Euclidian distance between the most distant claws on each CGC (Fig. 2F). This analysis resulted in an average distance between claws of 45.3 ± 2.2 μm (*n* = 35) for unbranched dendrites. However, on each branched dendrite, the inter-claw distance was only 22.1 ± 3.3 μm (*n* = 12). The average length of all CGC dendrites, measured between the soma and claw, was 23.3 ± 0.9 μm (*n* = 165) - consistent with a mean Euclidian distance between claws of ~45 μm and in agreement with the parasagittal dendrite coverage shown in Fig. 2B.

What was also clear from this analysis was that a large proportion of the total dendrite length was located within the claw-like structure of each dendrite (Fig. 2G). Figure 1F illustrates an individual dendrite claw at high-magnification, demonstrating the presence of the many small digits. This feature is responsible for the longer than expected total length of CGC dendrites. This feature was particularly evident for dendrites that contained displaced axons where half of the total dendrite length was contained within the claw. On average, 5.7 ± 0.6 μm of the dendrite was measured before the displaced axon and 15.9 ± 2.4 μm of dendrite was located between the displaced axon and the claw. But, the total length of the dendrite within the claw was 21.3 ± 2.0 μm. In contrast, the complexity of the claw region was reduced in dendrites more distant from the axon, where the average length of the dendrite found within the claw was only 12.1 ± 4.2 μm.

### Examining how the spacing of MF rosettes can influence sparse coding

The spacing between claws of an individual CGC helps determine the independence of MF inputs onto each CGC; a key determinant for sparse MF coding within the CGC layer. However, a central assumption of the sparse coding hypothesis is that the multiple rosettes associated with each MF axon are sufficiently distant from one another that activation of a single CGC by a single MF is simply not possible. To address this important aspect of MF expansion recoding we quantified the spacing of rosettes associated with individual MF axons (Fig. 3A). The 3D location of axonal swellings on individual axons was determined for 67 adult mouse MF axons identified from WGA-HRP stained fibres. Several obvious axonal swellings were identified on each MF (3 ± 0.3 rosettes per MF, *n* = 67) with a clear bimodal distribution of diameters (Fig. 3B). The smaller structures resembled boutons with a mean diameter of 1.6 μm, as determined from the peak of the Gaussian fit. The larger diameter structures resembled classical MF rosettes with a mean diameter of 7.1 μm that accounted for 95% of observations when considering the area under the fit. Mediodorsal and parasagittal coordinates were used to calculate Euclidian distances between boutons and rosettes. Using a cut-off diameter of 3 μm we estimated that putative axon boutons were spaced at an average distance of 8.8 ± 1.2 μm (n = 20) whereas, the MF rosette population was spaced at an average distance of 79.1 ± 14.2 μm (n = 137). However, the distribution of rosette spacings was very wide ranging from 4 μm to 1,167 μm. Using the mean distance between CGC claws of 45 μm as a threshold, we estimate that only 11 of the 67 analyzed MF axons would be unable to make multiple connections onto a single CGC. A direct comparison of claw spacing for individual CGCs and spacing of rosettes on each MF also revealed considerable overlap between these populations in the mediodorsal and parasagittal plane (Fig. 3C). A final comparison of cumulative probability plots constructed for CGC claw spacing and MF rosette spacing (Euclidian distances) demonstrated that only 20% of all rosettes were too distant to enable multiple connections from the same MF to a single CGC (Fig. 3D). Overall, these results seem to argue against the idea of independent MF inputs connecting to each dendrite of a single CGC.

To investigate the role of non-independent MF inputs in the cerebellar circuit, we ran simulations comparing a CGC network that received independent MF inputs to a network that allowed CGCs to be connected multiple times to the same MF. In each model a proportion of MFs was randomly activated and this was compared to the number of active CGCs. Both models contained 509 single compartment CGCs and 176 MFs with the upper and lower limits for dendrite length set by the 5 & 95% percentiles of our dendrite length measurements (3.4 to 28 μm). When the proportion of CGCs that fired was lower than the proportion of MFs activated at the start of the simulation, the network was performing ‘sparsification’. When each CGC dendrite was constrained to find connections with independent MFs then the network was found to be transforming the MF input into a sparser code, consistent with previous models^14^. When this constrain was removed we found that 38% of CGC dendrites connected to a MF that was already connected to the same CGC. In this shared input model, the simulation was also found to perform ‘sparsification’ but to a lesser extent than the previous independent MF model with the shared MF model more faithfully relaying the input pattern. However, one of the many simplifications that underlie this and many other cerebellar models is the assumption that all CGC inputs are treated as equal. Unfortunately, this assumption is likely to be at odds with both functional and anatomical data.

### CGC dendrite complexity depends upon axon location

**Figure 4:**
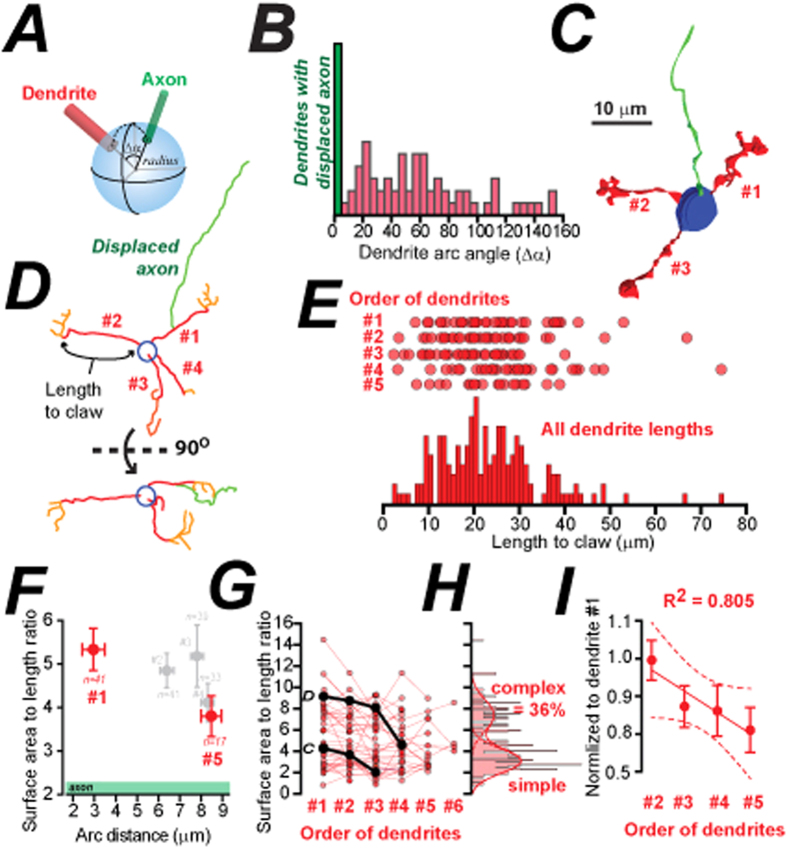
Establishing a relationship between dendrite complexity and axon location. (**A**) Cartoon illustration of the method used to classify CGC dendrite order. (**B**) Histogram of dendrite arc angles measured relative to the axon for all dendrites (red bars) including the dendrites with the displaced axon (green bar). (**C**) 3D reconstruction from an adult CGC with dendrite order indicated. (**D**) Illustration of the z-tracing method used to obtain length measurements for dendrite before the claw (red), dendrite within the claw (orange) and the axonal length (green) for adult CGCs. (**E**) Plots of Euclidian distance between claws for branched and unbranched dendrites. Each circle is a single measurement. (**F**) Plot of the average surface area to length ratio against the average arc distance for dendrite #1 to #5. Dendrite #1 and #5 have been highlighted in red as these show the greatest difference in both of these parameters. The error bars depict the SEM values. (**G**) Scatter plot of all surface area to length ratio estimates (red circles) ordered from dendrite #1 to #5. The black circles show the results for the adult CGCs shown in panels C and D of this figure. (**H**) Histogram of all surface area to length ratio estimates (red bars) with the multiple Gaussian fits (red line) to the two discernable peaks of this distribution superimposed. The area under the curve for the two Gaussians was used to estimate the proportion of simple and complex dendrites on adult CGCs. (**I**) Surface area to length ratio data has been normalized to the first dendrite for each cell and the average data has been fitted with a straight line. The results of this linear regression analysis (solid red line) and the 95% confidence levels for this fit (dashed red lines) are shown.

To systematically order the CGC dendrites with respect to the axon, we assumed the soma to be a sphere and calculated the arc distance using the Cartesian co-ordinates of the dendrite-soma and the axon-soma junction (Fig. 4A). There was a broad distribution of arc angles (Fig. 4B) with a mean value of 56 ± 5° (not including the zero angles for those occasions where the axon emerged from the dendrite). The adult CGC shown in Fig. 4C illustrates a simple cell with only three dendrites. The surface area of the claw region decreases in dendrites that are distant from the axon. On average the total length of dendrite #1 (dendrite length to claw + dendrite within the claw) was consistently the longest at 41.0 ± 13.3 μm (CV = 0.5, *n = *35) with a mean surface area of 221.0 ± 24.9 μm^2^ (CV = 0.7). Dendrite #2 was smaller (P = 0.02; paired Wilcoxon) with a total dendrite length of 30.0 ± 3.0 μm (CV = 0.6, n = 35) and a surface area of 163.9 ± 519.2 μm^2^ (CV = 0.7). Dendrites #3 and #4 were a similar size (dendrite #3 length of 34.2 ± 3.5 μm, CV = 0.6; surface area, 172.2 ± 24.2 μm^2^, CV = 0.8; n = 34; dendrite #4 length, 34.4 ± 3.8 μm, CV = 0.6, surface area, 147.5 ± 20.6 μm^2^, CV = 0.7, n = 28). However, dendrite #5 was consistently the smallest dendrite examined with a mean total dendrite length of 26.2 ± 3.1 μm (CV = 0.4, n = 13) and a mean surface area of 119.5 ± 21.1 μm^2^ (CV = 0.6). We have used the surface area to length ratio of each dendrite as an indicator of dendrite complexity (this measure does not require any identification of the claw region) and Fig. 4F compares the surface area to length ratio plotted as a function of the estimated arc distance calculated for dendrite 1 through to 5. The clearest difference in this data-set is observed between dendrite #1 and #5. The average surface area to length ration for dendrite #1 was 5.6 ± 0.5 (n = 41) compared to 2.1 ± 0.2 (*n* = 17) for dendrite #5 (P < 0.005; T-test) (). To estimate the proportion of complex versus simple dendrites on adult CGCs we examined the distribution of surface area to length ratio estimates across all dendrites. The surface area to length ratio for all dendrites is plotted in Fig. 4G. We have also illustrated the results of this analysis for the example cells shown in panel C and D of Fig. 4. The trend to progress from larger to smaller claws as a function of dendrite location is clear in these two examples. The histogram in Fig. 4H further illustrates, using multiple Gaussian fits, that 36% of CGC dendrites could be classified as complex when considering the surface area to length ratio.

### Developing models to explore the impact of morphological diversity

The imaging data demonstrates that the dendrite closest to the axon exhibits the highest degree of morphological complexity: a feature of CGC morphology that has not, to our knowledge, been previously reported. The increase in surface area of the claw will lead to a larger number of presynaptic release sites provided by the MF rosette arriving onto the dendrite closest to the axon. This feature should lead to an increased contribution from the MF rosette closest to the axon. In order to test this hypothesis we constructed three different CGC compartmental models. Model 1 was designed to study the impact of dendrite complexity on CGC function, whereas model 2 examines the influence of dendrite branching, and model 3 explores the impact of axonal displacement along the primary dendrite. Volume and length measurements derived from 3D models were used to estimate the average diameter of each cylindrical compartment in the CGC model. For example, the length of the recovered ascending axon in mature CGCs (≥P30) was 176 ± 331 μm (n = 41), with a total volume of 52.5 ± 12.9 μm^3^. The axonal compartment has, therefore, been represented by a cylinder of 176 μm length and 0.6 μm diameter.

**Figure 5:**
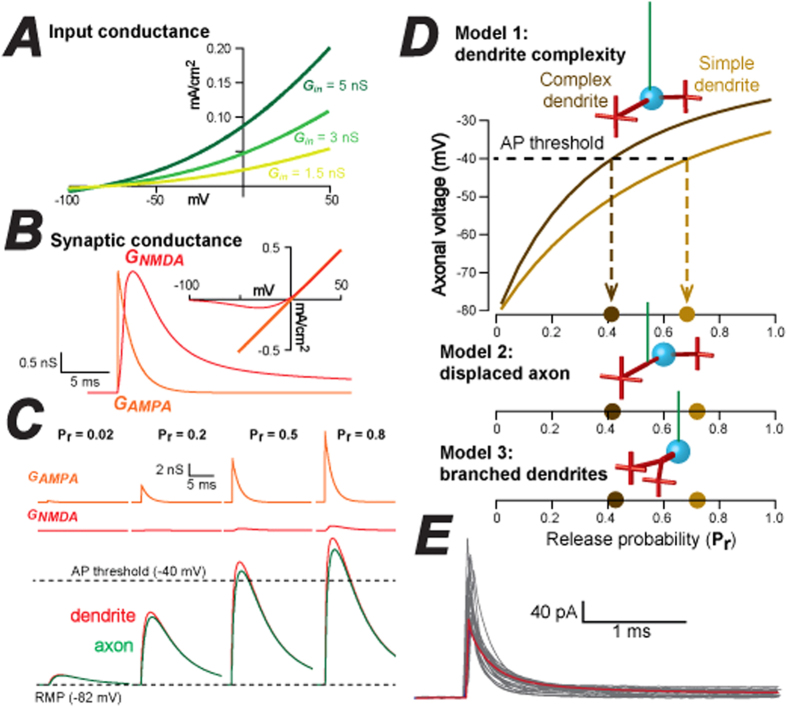
Demonstration that MF inputs onto complex dendrites have a greater impact on CGC excitability. (**A**) A series of three current-voltage (I-V) relationships used to simulate different CGC input conductance levels using the Goldman-Hodgkin-Katz equation to produce open rectification of the input conductance. (**B**) Illustration of the conductance waveform profiles used to mimic AMPA-type (orange trace) and NMDA-type (red trace) postsynaptic conductance changes in CGC models. The I-V relationship for the AMPA and NMDA component of the postsynaptic response are also shown. A linear Ohmic relationship is used to mimic the AMPA-type synapse whereas a more complex Boltzmann function is used to mimic the voltage-dependent Mg^2+^ block that is characteristic of an NMDA-type postsynaptic response. (**C**) Illustration of how increasing the release probability leads to a larger AMPA and NMDA-component of the postsynaptic conductance change and therefore, influences the magnitude of the EPSP using a single synapse on CGC model 1. The bottom traces compare the voltage change produced at the dendrite (red) and axon (green), illustrating the small degree of filtering introduced by CGC morphology. (**D**) Plots illustrating the relationship between the peak axonal voltage and the release probability at a synapse located on the simple and complex dendrite of model 1, 2 and 3. The dashed lines indicate the voltage at which an action potential (AP) would be initiated in a typical CGC indicating how AP threshold would be reached at a lower release probability for a synapse located on the complex dendrite compared to a synapse on the simple dendrite. (**E**) Simulated capacitance transient from model 1 (red trace) is superimposed onto all capacitance transients recorded from the cerebellar granule cells used for morphological analysis in this study. Note the similarity.

The outward rectification that characterizes the input conductance of adult CGCs^28^ was simulated using the Goldman-Hodgkin-Katz (GHK) equation (Fig. 5A). The magnitude of the GHK leak conductance was based upon whole-cell voltage-clamp measurements obtained from adult CGCs. The synaptic conductance waveforms used to simulate glutamate release at individual release sites onto the CGC dendrites were based on measurements of spontaneous AMPA/NMDA receptor-mediated conductance changes in the presence of TTX (Fig. 5B). Unitary synaptic conductance waveforms were introduced onto individual digits within the claw region and voltage changes were monitored in all compartments. Their simple morphology has led to the suggestion that CGCs behave as a single electrotonic compartment^29^. However, it would appear that CGC shape introduces a small degree of EPSP filtering (Fig. 5C) in all three models (see Fig. 5D), once again arguing against a simple single compartmental model^30^,^31^. As a simple verification of the models used in this study we simulated a capacitance transient from each model and compared this to the actual transients recorded from all CGCS used for morphological analysis (Fig. 5E).

### Dendrite complexity will influence MF selectivity when inhibition is low

**Figure 6:**
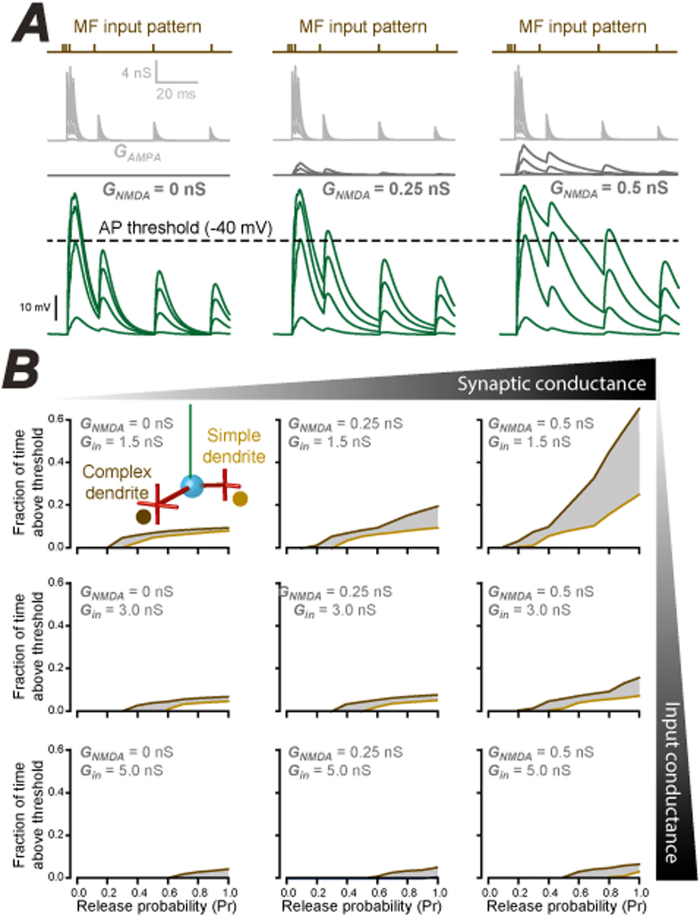
Influence of dendrite complexity, synaptic drive and inhibition on MF coding. (**A**) The MF input pattern chosen in these simulations (brown top traces) was based upon *in vivo* measurements of MF bursting patterns (Rancz *et al*. 2007)^10^. The resulting simulation of the AMPA and NMDA conductance (grey traces) is shown with the peak NMDA component increasing from 0 to 0.25 and 0.5 nS (left to right). The bottom traces (green) show the resulting voltage responses indicating the time spent above the AP threshold for CGCs (dashed line). (**B**) A series of plots quantifying the fraction of time that the membrane voltage spends above AP threshold when the MF input pattern (see Fig. 4) is delivered to either the simple (light brown) or complex (dark brown) dendrite at increasing release probabilities. The top left panel illustrates the results of this simulation when the input conductance of the CGC is set to 1.5 nS and the peak conductance of the NMDA component of the synaptic response is set to 0 nS. The NMDA component of the synaptic conductance is then increased from 0 to 0.25 and 0.5 nS from left to right, whereas the CGC input conductance is increased from 1.5 to 3.0 and 5.0 nS as the panels move from top to bottom. These results were obtained for model 1, with identical results obtained for model 2 and 3.

The arrival of MF information at either simple or complex dendrites was examined as release probability (Pr) was increased. The superimposed traces shown in Fig. 6A illustrate the synaptic conductance changes that take place in the complex dendrite of model 1 (grey traces) and the resulting voltage change in the axon initial segment (green traces). In these examples, the release probability during patterned MF input was set at 0.02, 0.2, 0.5 and 0.8. In the set of left-hand traces the peak NMDA conductance was 0 nS, whereas in the middle traces the NMDA peak conductance was set at 0.25 nS, and increasing to 0.5 nS for the right-hand traces. As expected, given the small size of individual synaptic responses at individual sites, a high release probability of at least 0.5 was required to reach AP threshold during stimulation of a single MF input. However, there was a very obvious increase in the time spent above action potential threshold as the relative contribution of the synaptic NMDA receptor-mediated conductance was increased (left to right). As illustrated in Fig. 6B, the same MF input pattern was then introduced into either the simple or complex dendrites. The fraction of time spent above AP threshold at the axon initial segment was measured in response to increased synaptic conductance strength and input conductance. As the NMDA component of the synaptic response was increased, the difference between the simple and complex dendrites became greater (Fig. 6B). The non-linearity of this relationship was due to the voltage-dependence of the NMDA component. However, the difference between MF inputs arriving on the simple and complex dendrites (grey shaded area) became smaller as the input conductance of the CGC model was increased to mimic enhanced tonic inhibition. Therefore, differences in dendritic complexity will have a greater influence on CGC output when the input conductance of CGCs is low and the contribution of the NMDA receptor mediated component of the synaptic response is high. This behavior was observed for all three CGC models indicating that selectivity of MF inputs occurs irrespective of dendrite branching or the presence of a displaced axon. In summary, we have described several unusual features of CGC morphology that could result in different MF inputs making very different functional contributions to CGC output and we describe how these influences could be regulated by changes in inhibition. Our observations predict that CGC shape will influence synaptic integration within the CGC layer and highlight a need to incorporate these features into more comprehensive models of cerebellar function.

## Discussion

Whole-cell recordings in acute slice preparations indicated that variability in CGC size could impact on the timing and reliability of MF-evoked APs. 3D models of individually labelled CGCs and analysis of MF rosette spacing has highlighted the ability of the CGC layer to encode MF information with greater reliability than previously expected. We have shown that the CGC dendrites closest to the axon are more complex in structure and we propose that CGCs can preferentially select for MF information arriving onto the claw of these more complex dendrites. The selectivity introduced by the presence of simple and complex dendrites was accentuated when the NMDA component of the excitatory synaptic drive was enhanced. This raises the possibility that long-term plasticity at MF → CGC connections^6^,^32^ could emphasise the selection of sensory input arriving onto the dendrite closest to the axon, or may even be responsible for the observed morphological differences due to activity-dependent structural changes^33^. Finally, the model predicts that simply raising the magnitude of the CGC input conductance^34^ will counteract the influence of dendrite complexity suggesting that the sensitivity of CGCs to these differently weighted MF inputs will be modulated by factors such as tonic inhibition.

### CGC shape is known to influence MF precision

The experiments we describe in Fig. 1 demonstrate how CGC morphology could influence AP timing. Previous studies of CGC excitability, using both acute slice preparations^29^ and *in vivo* recording^35^, have stressed the importance of high temporal fidelity for MF → CGC connections. Many variables are known to impact upon AP timing within CGCs such as differences in release probability at individual MF → CGC synapses^21^, glutamate spill-over between MF release sites^19^, and even time-dependent changes in CGC input conductance^36^. CGC morphology is rarely considered in relation to the cerebellar circuit^37^ but Sultan *et al*. (2001)^26^ did discuss how mediolateral spread of the ascending CGC axon will introduce a >200 μs smearing in the arrival of parallel fibre information onto Purkinje cells due to the slow conduction velocity of these long thin fibres^38^. Therefore, the temporal precision of information transfer along the MF → CGC → Parallel fibre pathway will be enhanced by the narrow spread of the ascending CGC axon that we describe in Fig. 2A and B. Such restricted mediolateral spread is typical of an “anisotropic” structure like the cerebellum. It mirrors, for example, the climbing fibre input to the Purkinje cell population^24^. However, we now show that CGC dendrite topology is also anisotropic with each of the 5 or more dendritic claws extending from a single CGC exhibiting restricted mediolateral spread and greater dendrite coverage in the parasagittal plane.

We find axon displacement to be present in 40% of adult CGCs. Axon displacement was originally described by a student of Cajal in the early 20^th^ century and was evident in over half of the CGCs drawn by Cajal^39^. Similar observations have made in pyramidal cells^40^, hippocampal interneurons^41^, neuroendocrine cells^42^, and mid-brain dopamine neurons^43^. The functional significance of axon displacement was first examined for dopamine neurons^44^ leading to the suggestion that excitatory inputs arriving closer to the displaced axon were “privileged” in their ability to excite the neuron. This was later confirmed for hippocampal pyramidal neurons with the demonstration that APs elicited following glutamate uncaging onto the dendrite carrying the displaced axon exhibited both a shorter latency as well as lower activation thresholds^45^. A similar phenomenon is likely to occur for CGCs, in part explaining the variability that is characteristic of the timing of MF-evoked APs.

### The importance of considering CGC claw spacing in relation to MF rosette spacing

A single MF axon generates 3–20 large axon terminal rosettes within a small region of the cerebellar cortex^39^,^46^ with each rosette making connections with the claw endings of 15 dendrites^26^,^47^,^48^. The distance between CGC claws is a key parameter when discussing issues such as expansion recoding at the MF → CGC pathway. The assumption that CGC dendrite claws are at least 10 μm apart ensures that the 15 claws connected to a single rosette will arise from 15 independent CGCs. Our quantitative treatment of CGC morphology confirms that this is likely to be the case when considering the claws of unbranched dendrites, but for branched dendrites we do occasionally observe spacing between claws of less than 10 μm (Fig. 2F). There is no available corroborative anatomical or functional evidence to suggest that the two claw endings provide by a branched dendrite are indeed connected to a common MF rosette. However, dendrite branching is clearly a prevalent feature of adult CGCs (40% in this study) that increases the average number of MF rosettes connected to a single CGC from 4 to 5; with as many as 8 MFs connected to the most branched CGCs. To what extent the information storage capacity of the MF → CGC pathway is altered by dendrite branching will also depend on the independence of each MF rosette. Cajal illustrates two examples of a single MF input generating two MF rosettes that are connected to a single CGC^39^. If this were a common feature of MF → CGC connectivity then expansion recoding could be far less pronounced than currently assumed (**see** Fig. 3). Livet *et al*. (2007) also documented a single CGC connected to two rosettes that were genetically shown to originate from the same MF^49^. Due to an absence of quantitative data, models of the cerebellar circuit do not incorporate this feature into the MF → CGC pathway^1^,^2^,^3^,^14^. It has previously been shown that the average distance between neighboring MF rosettes is 60 μm in the parasagittal plane compared to only 20 μm in the mediolateral plane^26^. We have excluded smaller sized MF boutons from our analysis and concentrated on quantifying the distance between the larger rosettes that are known to be present within cerebellar glomeruli.

Our quantification of CGC dendrites demonstrates that the average distance between claws ranges from 20 to 80 μm for unbranched dendrites in the parasagittal plane. It is also clear, from the restricted mediolateral spread of CGC dendrites, that shared MF inputs would be less common in this axis. This could favor selecting for MF inputs that contribute to a tidal-wave like mechanism, as suggested by theoretical reasoning^50^,^51^. In contrast, the wide parasagittal coverage of CGC dendrites makes shared MF inputs a likely feature of MF → CGC coding along the parasagittal axis. Our model then demonstrates how shared MF input would reduce so called “sparcification” of sensory input onto the CGCs with clear implications for expansion recoding.

### Integration of multimodal MF sensory input by CGCs

We show that more complex dendrites are found nearest the axon (see Fig. 4). The greater surface area to length ratio of the claw on this dendrite means that the excitatory drive onto this dendrite could be enhanced. This feature could contribute to the diversity of synaptic behaviours that has been reported at MF → CGC connections^21^. This study by Chabrol *et al*. (2014) identified strong and weak MF inputs to CGCs that were capable of producing short and long latency responses similar to those described in Fig. 1. However, it is not currently clear to what extent a single CGC is required to select for MF inputs that code for different aspects of the sensory world. There is functional evidence in cats^35^ for convergence of different types of MF input onto a single CGC. Similarly, in the cerebellum like circuit of mormyrid fish Sawtell (2010)^52^ identified a subset of CGCs that responded to sensory and motor information conveyed by different MF inputs. Multimodal integration of auditory, visual and somatosensory input within a single CGC has also been elegantly demonstrated in the rodent cerebellum^53^, and anatomical tracing^48^ also demonstrates that subsets of CGCs can receive MF input from both motor and sensory pathways. There is also experimental evidence to suggest that CGCs respond differently according to the nature of the MF input^54^. Somatosensory stimulation (whisker or forepaw) results in bursts of MF inputs that translate reliably into synaptic responses^10^,^35^, whereas proprioceptive signals such as body position appear to respond with more graded tonic changes in synaptic response over a range of stimulus intensities^52^,^55^. Our observation that the ability of the MF → CGC pathway to select for particular MF inputs is enhanced by differences in dendrite complexity could have important implications for multimodal integration as information arriving onto complex dendrites could be preferentially selected over weaker inputs.

### Implications of CGC dendrite heterogeneity for recoding of MF information

This theoretical study demonstrates how increasing CGC input conductance could effectively reduce any differences in excitability introduced by the presence of complex and simple dendrites. Experimental data has shown how CGC input conductance can be be increased by activation of extrasynaptic GABA_A_ receptors following raised ambient GABA levels^11^, or by an increased contribution from potassium leak channels^56^ following the action of neuromodulators^57^. Regardless of the mechanism, dendrite heterogeneity will be less important when CGC input conductance is high and the simpler integrate and fire behaviour of CGCs will be less able to select for MF inputs arriving onto complex dendrites.

## Methods

### Cerebellar granule cell preparation

Male C57Bl/6 J mice (aged 2–3 months) were sacrificed in accordance with UK Home Office regulations (approved by the Imperial College Ethical Review Committee) by cervical dislocation followed by decapitation and the cerebellum detached from the forebrain. Acute slice preparations were cut parasagitally from the vermis at a thickness of 250 μm at ~4 °C in a glycerol-based artificial cerebro-spinal fluid (ACSF) (in mM): 2.5 KCl, 1.25 NaH_2_PO_4_, 26 NaHCO_3_, 250 Glycerol, 5 MgCl_2_, 1 CaCl_2_, 11 Glucose, bubbled with 95%O_2_/5%CO_2_ (pH 7.4). Once slicing was complete slices were heated to 37 °C for 40 minutes while the glycerol-based ACSF was slowly exchanged for control ACSF (in mM: 125 NaCl, 2.5 KCl, 2 CaCl_2_, 2 MgCl, 1.25 NaH_2_PO_4_, 26 NaHCO_3_, 11 glucose, pH 7.4 when bubbled with 95%O_2_/5%CO_2_) and cooled to room temperature before recording^34^. Glass electrodes were pulled to a final resistance of 8–9 MΩ from borosilicate glass capillaries and filled with an internal solution that contained (in mM) 145 K-gluconate, 4 NaCl, 5 KCl, 0.5 CaCl_2_, 10 HEPES, 5 EGTA, 4 Mg-ATP, 0.3 Na-GTP (pH 7.3) and 1.5 mg/ml biocytin (Invitrogen^®^, USA). Fixation of slices in 4% PFA was performed following successful whole-cell recording. Cells were permeabilized with 0.2% Triton-X at room temperature for 1–2 hours and non-specific binding was blocked with 5% donkey serum in PBS. A 1:200 dilution of streptavidin-Alexa555 (Invitrogen^®^, USA) was then conjugated to the biocytin and slices were washed in PBS and mounted in Vectashield^®^eH-1000 (refractive index = 1.44) on glass slides with Menzel cover glasses (0 thickness).

### Electrophysiological recordings

CGCs were visualized using a fixed-stage upright microscope (BX51W1, Olympus or Scientifica) fitted with a high numerical aperture water-immersion objective and a digital camera. The recording chamber was continuously perfused with solutions that entered the bath via a gravity perfusion system at a rate of 3 ml/min. Fine and course movement of the pipettes were controlled by micromanipulators (PatchStar, Scientifica) mounted upon a fixed platform. The current and voltage output of the Axoclamp 700B amplifier (Molecular Devices; Foster City, CA) was filtered at 10 kHz (−3 dB, 8-pole low-pass Bessel) and digitized at 50 kHz using a National Instruments digitization board (NI-DAQmx, PCI-6052E; National Instruments, Austin, Texas). Data acquisition was performed using WINWCP (Version 4.1.2) and WINEDR (Version 3.0.9) provided by John Dempster (John Dempster; University of Strathclyde, UK). Total membrane capacitance (*C*_*m*_) was calculated offline from voltage-clamp data according to:


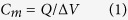


where, Q is the charge transfer measured from the current record during a hyperpolarising 10 mV step in the amplifiers command voltage (∆V). Q was calculated from an average of 20 consecutive current transients recorded immediately following breakthrough into whole-cell configuration at a command voltage of −60 mV. Action potentials were elicited from CGCs by direct somatic current injection or following MF electrical stimulation.

### Confocal imaging

Following electrophysiological recording, individual CGCs were imaged using a Zeiss LSM 510 CLSM microscope, with a He-Ne 543 nm laser and filter settings optimised for an Alexa-555 emission peak at 565 nm. A 40x oil-immersion objective was used with a numerical aperture (*NA*_*obj*_) of 1.3. The emission wavelength (λ_O_) was 700 nm, and the refractive index (η) of the immersion oil was 1.516. Given these parameters, the optical section thickness was set at 1.02 μm. The confocal pinhole was set to one Airy unit and scan speeds were typically set to 9.3 seconds/slice with the detector gain, amplifier gain and amplifier offset of the photomultiplier chosen to avoid saturation of the fluorescent signal. This consideration meant that the CGC soma (5–8 μm in diameter) was imaged at a different setting to the dendrites (1–2 μm diameter) and axons (<1 μm in diameter). To increase the signal to noise ratio, frame averaging was applied to all images such that each scan was repeated four times to enable pixel-by-pixel comparisons of fluorescence. Deconvolution analysis was used to correct for diffraction problems and blurring of the confocal image. *ImageJ* software^53^ was used to perform deconvolution analysis. For each 2D image in the stack, a theoretical point spread function (PSF) was calculated using the *DiffractionPSF 3D* plug-in, given the numerical aperture of the lens, the refractive index of the immersion medium, pixel size and image size in x, y and z direction. The calculated PSFs together with the 2D image stacks for each CGC were uploaded into the software. Deconvolution was carried out using the plug-in *Iterative_Decolvolve_3D*, which uses a DAMAS algorithm to perform the deconvolution analysis^54^. The deconvolved image showed improved image quality and reduction in blurring and diffraction-generated artefacts.

### 3D Reconstructions

Each stack of consecutive optical sections was uploaded into *Reconstruct3D*^10^. Morphological parameters such as surface area, volume, length and distance between axon and dendrite were extracted from the 3D reconstructions. Surface area of a given structure was calculated by summating the circumference of the structure across all optical sections multiplied by the optical section thickness. The axon to dendrite distance was measured by assuming the soma to be a sphere and calculating the arc radius between two points: the dendrite-soma junction and the axon-soma junction. The Cartesian coordinates for the dendrite-soma junction and axon-soma junction were extracted from the 3D reconstructions. These coordinates were then transformed into a spherical coordinate system and the angle subtended by these two points was calculated. The arc length (Δσ) was calculated according to:


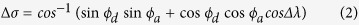


Where ϕ_d_is the angular distance between the horizontal plane and point d. ϕ_a_ is the angular distance between the horizontal plane and point a. Δλ is the difference in the angular distances between vertical planes, points d and a, respectively. Dendrites were then ordered based on their proximity to the axon - dendrite #1 being closest to the axon. In the case where the axon was found to leave from a dendrite, this dendrite was labelled dendrite #1.

### Analysis of MF rosette spacing

This data-set was obtained from a previous study^26^ in which cerebellar white matter injection in the parasagittal part of the vermis was used to label MFs in the adult mouse with wheat germ agglutinin conjugated to horseradish peroxidase (WGA-HRP). Individual MFs were visualized with a 100 X oil immersion objective (NA 1.3) on a Zeiss Axiophot microscope. The Eutectic neuron tracing system (version 4.1: Raleigh, NC, USA) was used to trace the axon trajectory and mark the location of rosettes and boutons. The size of these axonal swellings was estimated from the diameter of an adjustable circle fitted to the image.

### NeuroConstruct simulations

The neuroConstruct^58^ program was used to incorporate network parameters into models of the granule cell layer that were then simulated within NEURON^59^. Simulations were adapted from a published model constructed by Billings *et al*. (2014) 14, to investigate sparse encoding in the cerebellum. This was a scaled down model of the cerebellar granule cell layer comprising 509 single compartment granule cells and 176 single compartment mossy fibres. The structure of the model remained largely unchanged from the published version but some parameters of the model were altered to reflect the new experimental data. Most importantly, the original model specified that CGC dendrites should not exceed 15 μm, citing the measurements by Palkovits *et al*. (1972)^23^. However, new morphological data presented in this study showed that dendrite length frequently exceeded this value. Therefore the 5th and 95th percentiles were taken from the measured dendrite lengths and set as the upper and lower limits of dendrite lengths allowed in the model. This gave a range from 3.4 μm to 27.9 μm. Connections were then randomly within this specified range of dendrite lengths. However, in our modified model, CGCs could make multiple network connections to the same MF resulting in 38% of CGC dendrites connecting to a mossy fibre that had previously been connected to the same cell. MFs were activated using a random spike generator in NeuroConstruct and the simulation was repeated with increasing numbers of MFs being activated each time. Data generated by the simulations was analysed in Matlab to determine how many cells from each population spent time above threshold and so were considered to have fired.

### NEURON simulations

The software program NEURON was used to simulate CGC voltage changes in response to MF input. Multi-compartment cable equations were used to model membranes as equivalent electrical circuits and allow for different compartments within the simulated neuron to have different radii and lengths as well as different membrane conductance properties. The outward rectification that characterizes the input conductance of adult CGCs was simulated using the Goldman-Hodgkin-Katz (GHK) equation, which models the diffusion of ions through a uniformly permeable membrane. The GHK current equation^60^, calculates the net current flow *I*_*X*_ of a membrane-permeable ion *X* per unit area of membrane, as follows:


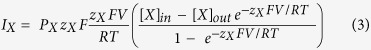


where 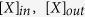 are the intracellular and extracellular concentrations of ion *X, z*_*X*_ the valency of *X, P*_*X*_ the membrane’s permeability to *X, V* the voltage across the membrane, *F* the Faraday constant, *R* the gas constant and *T* the temperature.

The density of MF release sites was taken as 3 release sites per μm^2^ 61 and the release probability was increased by simply altering the number of active release sites until the maximum was reached. The postsynaptic current that results from neurotransmitter release at time *t*_*s*_, for *t* ≥ *t*_*s*_ was determined by:


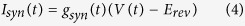


where 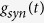 is the conductance change in membrane due to the effect of transmitter in the postsynaptic receptors, 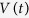 is the postsynaptic membrane potential and 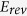 is the reversal potential of the ion channels that mediate the synaptic current^62^. Excitatory synaptic conductance changes were inserted onto the digits with the dendrite claw. The synaptic mechanism was modified^63^ to fit our experimental data. The AMPA-type glutamate receptor contribution was modelled as an alpha function^64^,^65^:


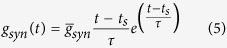


while the NMDA receptor was based on two-state kinetics scheme 66 of the form:


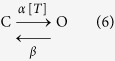


where *O* and *C* are the two available states for the receptor, open and closed, respectively, *α* and *β* are voltage-independent forward and backward rate constants and *[T]* is the transmitter concentration in the synaptic cleft, which regulates the transition between the two possible states. The synaptic conductance was represented as:


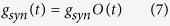


where 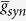 is the maximal unitary synaptic conductance. 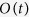
is defined as the fraction of the receptors in the open state and it is described by the following first-order differential equation:


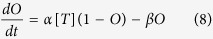


A voltage dependent term, the magnesium block (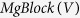) was included in the NMDA mechanism^29^:


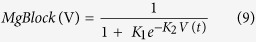


where *K*_1_ and *K*_2_ are the parameters that determine the voltage dependence of the block^66^:


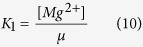


where 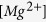 varies from 1 to 2 mM in physiological conditions and μ is a constant. The synaptic response also included a frequency dependent plasticity rule to account for frequency dependent synaptic depression^29^,^55^. NEURON’s vector stream of events mechanism (vecevent.mod) was used to specify the timing and magnitude of each synaptic conductance. The SEClamp feature was used within NEURON to simulate capacitance transients. The data generated from simulations in NEURON were saved in ASCII format and imported into Matlab (Mathworks) to measure EPSP waveforms and the fraction of time spent above threshold. Data was further analyzed and plotted in Origin before final figures were prepared using Illustrator.

## Additional Information

**How to cite this article:** Houston, C. M. *et al*. Exploring the significance of morphological diversity for cerebellar granule cell excitability. *Sci. Rep.* **7**, 46147; doi: 10.1038/srep46147 (2017).

**Publisher's note:** Springer Nature remains neutral with regard to jurisdictional claims in published maps and institutional affiliations.
